# Three-dimensional printing technology for localised thoracoscopic segmental resection for lung cancer: a quasi-randomised clinical trial

**DOI:** 10.1186/s12957-020-01998-2

**Published:** 2020-08-24

**Authors:** Yangming Chen, Jiguang Zhang, Qianshun Chen, Tian Li, Kai Chen, Qinghua Yu, Xing Lin

**Affiliations:** 1 Shengli Clinical Medical College, Fujian Medical University, Fuzhou, 350001 China; 2 Department of Thoracic Surgery, Fujian Provincial Hospital, Fuzhou, 350001 China; 3 Department of Radiology, Fujian Provincial Hospital, Fuzhou, 350001 China

**Keywords:** 3D printing technique, Position, Ground glass nodule, Thoracoscopic segmentectomy

## Abstract

**Background:**

Three-dimensional (3D) computed tomography (CT) reconstruction technology has gained attention owing to its potential in locating ground glass nodules in the lung. The 3D printing technology additionally allows the visualisation of the surrounding anatomical structure and variations. However, the clinical utility of these techniques is unknown. This study aimed to establish a lung tumour and an anatomical lung model using 3D printing and 3D chest CT reconstruction and to evaluate the clinical potential of 3D printing technology in uniportal video-assisted thoracoscopic segmentectomy.

**Methods:**

Eighty-nine patients with ground glass nodules who underwent uniportal video-assisted thoracoscopic segmentectomy were classified into the following groups: group A, lung models for pre-positioning and simulated surgery that were performed with 3D chest CT reconstruction and 3D printing, and group B, patients who underwent chest CT scans with image enhancement for 3D reconstruction. The differences in the surgery approach transfer rate, surgical method conversion rate, operative time, intraoperative blood loss, and postoperative complication rate were compared between the two groups.

**Results:**

Between groups A and B, there were significant differences in the approach transfer rate (0% vs.10.5%, *p* = 0.030), operative time (2.07 ± 0.24 h vs. 2.55 ± 0.41 h, *p* < 0.001), intraoperative blood loss volume (43.25 ± 13.63 mL vs. 96.68 ± 32.82 mL, *p* < 0.001) and the rate of surgical method conversion to lobectomy (0% vs. 10.5%, p < 0.030). In contrast, there was an insignificant difference in the postoperative complication rate between groups A and B (3.9% vs. 13.2%, *p* = 0.132).

**Conclusions:**

3D printing technology facilitates a more accurate location of nodules by surgeons, as it is based on two-dimensional and 3D image-based findings, and therefore, it can improve surgical accuracy and safety. This technique is worth applying in clinical practice.

## Background

According to the China National Health and Wellness Committee’s third survey, cancer has become the leading cause of death among urban residents in China, with lung cancer ranking first [1]. With the widespread use of low-dose computed tomography (CT) scans, many lung ground glass nodules (GGNs) have been discovered at an early stage and are monitored for their potential malignant transformation [2, 3]. Currently, surgery remains the main method of treatment for GGN. Thoracoscopic surgery is the standard treatment for early-stage lung tumours. The advantages of this technique include minimal trauma and a rapid rate of postoperative recovery, and thus, it is widely recognised and applied by thoracic surgeons [3, 4]. Anatomical segmentectomy has been proven to be an effective surgical treatment that can retain as much of the lung tissue as possible and yet achieve an oncological effect that is comparable to the oncological effect achieved with lobectomy [5]. In addition to the location of the target lesion, the definition of intersegmental segmentation is critical for anatomical segmentectomy. Recently, three-dimensional (3D) CT reconstruction technology has received a lot of attention as it can facilitate in locating the position of lung GGNs [4]. With the development of 3D printing technology and its introduction into the field of surgery, the relationship between lung anatomy and lung tumour can be visualised preoperatively, which can help the surgeon to determine the specific location of the lesion, the surrounding anatomical structure, and characteristic variations; simulate surgical procedures; and explore the optimal surgical path to reduce the rate of surgery, operative time, and intraoperative blood loss. This study aimed to analyse the potential application of 3D chest CT reconstruction combined with 3D printing technology in uniportal video-assisted thoracoscopic segmentectomy.

## Methods

### Ethics statement and patients

All procedures were carried out in accordance with the principles of the Declaration of Helsinki. The study was approved by the Clinical Research Ethics Committee at the Fujian Provincial Hospital, Fujian Province, China (K2015-022-01, September 30, 2015). Informed consent was obtained from all patients included in this study.

### Inclusion and exclusion criteria

Patients whose chest CT scans showed pulmonary GGNs and who underwent segmentectomy at Fujian Provincial Hospital between March 2016 and September 2018 were enrolled in this study. The inclusion criteria were a single lesion located in the lateral one third of the lung parenchyma with a diameter ≤ 2 cm, and according to the indications for segmental resection in the National Comprehensive Cancer Network (NCCN) guidelines, an indication for uniportal video-assisted thoracoscopic segmentectomy. On the contrary, the exclusion criteria were central lesions with a tumour-to-intersegmental fissure distance < 2 cm, multiple lesions, patients who do not meet the indications for segmental resection based on the NCCN guidelines, and general conditions that do not allow surgery or preoperative examinations suggesting distant metastasis (see study flow diagram).

### General information

All patients admitted to the hospital for surgical treatment, excluding those with poor surgical tolerance, distant metastasis, stroke sequelae, severe lung ventilation dysfunction, and other conditions, routinely undergo pulmonary function tests, cardiac ultrasound, total abdominal colour Doppler ultrasound, cranial magnetic resonance imaging, and whole-body bone scanning. All patients who met the enrolment criteria were included and randomly classified into the experimental group A and control group B using the coin method. This implies that, after obtaining informed consent from each patient who met the admission criteria, a coin was tossed to determine group allocation—if “heads”, patients were assigned to the experimental group A, and if “tails”, patients were assigned to the control group B. All patients were blinded to their group allocation.

In total, 101 individuals met the inclusion criteria. Among them, 4 patients refused to participate in the experiment, 2 patients were excluded due to poor physical condition, 1 patient cancelled the surgery unexpectedly, and after allocation, 5 patients were indicated to have benign lesion based on the frozen section examination. These 5 patients, therefore, exclusively underwent wedge resection. For the experimental group A, 3D chest CT reconstruction combined with the 3D printing technique was used in simulating a model to analyse the anatomical relationship and variations. For control group B, only a 3D CT scan was performed. In total, 89 patients were included in this study; 51 and 38 patients were assigned to the experimental group A [21 men and 30 women, aged 43–80 years, mean age 62 years; 17 patients were smokers (all men)] and control group B [14 men and 24 women, aged 40–81 years, mean age 61 years; 9 patients were smokers (all men)], respectively. No significant differences were observed in the general characteristics between the two groups (Table 1).

**Table 1:** Characteristics of the study cohort

	Experimental group A (*n* = 51)	Control group B (*n* = 38)	Total (*n* = 89)	*T*/ *χ*^2^ value	*p* value
Age [year, average age (± SD)]	60.73 ± 5.643	61.61 ± 6.445	61.10 ± 5.979	-0.685	0.495
Gender					
Male [no. of cases (%)]	21 (41.2)	14 (36.8)	35 (39.3)	0.171	0.679
Female [no. of cases (%)]	30 (58.8)	24 (63.2)	54 (60.7)		
History of smoking					
Smoker [no. of cases (%)]	17 (33.3)	11 (28.9)	28 (31.5)	0.194	0.659
Non-smoker [no. of cases (%)]	34 (66.7)	27 (71.1)	61 (68.5)		
Position (no. of cases, proportion %)					
Left lung	18 (35.3)	13 (34.2)	31 (34.8)	0.011	0.915
Right lung	33 (64.7)	25 (65.8)	58 (65.2)		
LS1 + 2	3 (5.9)	2 (5.3)	5 (5.6)	0.829	1.000
LS1 + 2 + 3	3 (5.9)	3 (7.9)	6 (6.7)		
LS4 + 5	3 (5.9)	2 (5.3)	5 (5.6)		
L3	2 (3.9)	1 (2.6)	3 (3.4)		
L6	2 (3.9)	2 (5.3)	4 (4.5)		
L basal segment	5 (9.8)	3 (7.9)	8 (9.0)		
RS1	6 (11.8)	4 (10.5)	10 (11.2)		
RS1 + 2	7 (13.7)	5 (13.2)	12 (13.5)		
RS3	3 (5.9)	2 (5.3)	5 (5.6)		
RS6	5 (9.8)	3 (7.9)	8 (9.0)		
R basal segment	12 (23.5)	11 (28.9)	23 (25.8)		

### 3D reconstruction and 3D printing

**Fig. 1:**
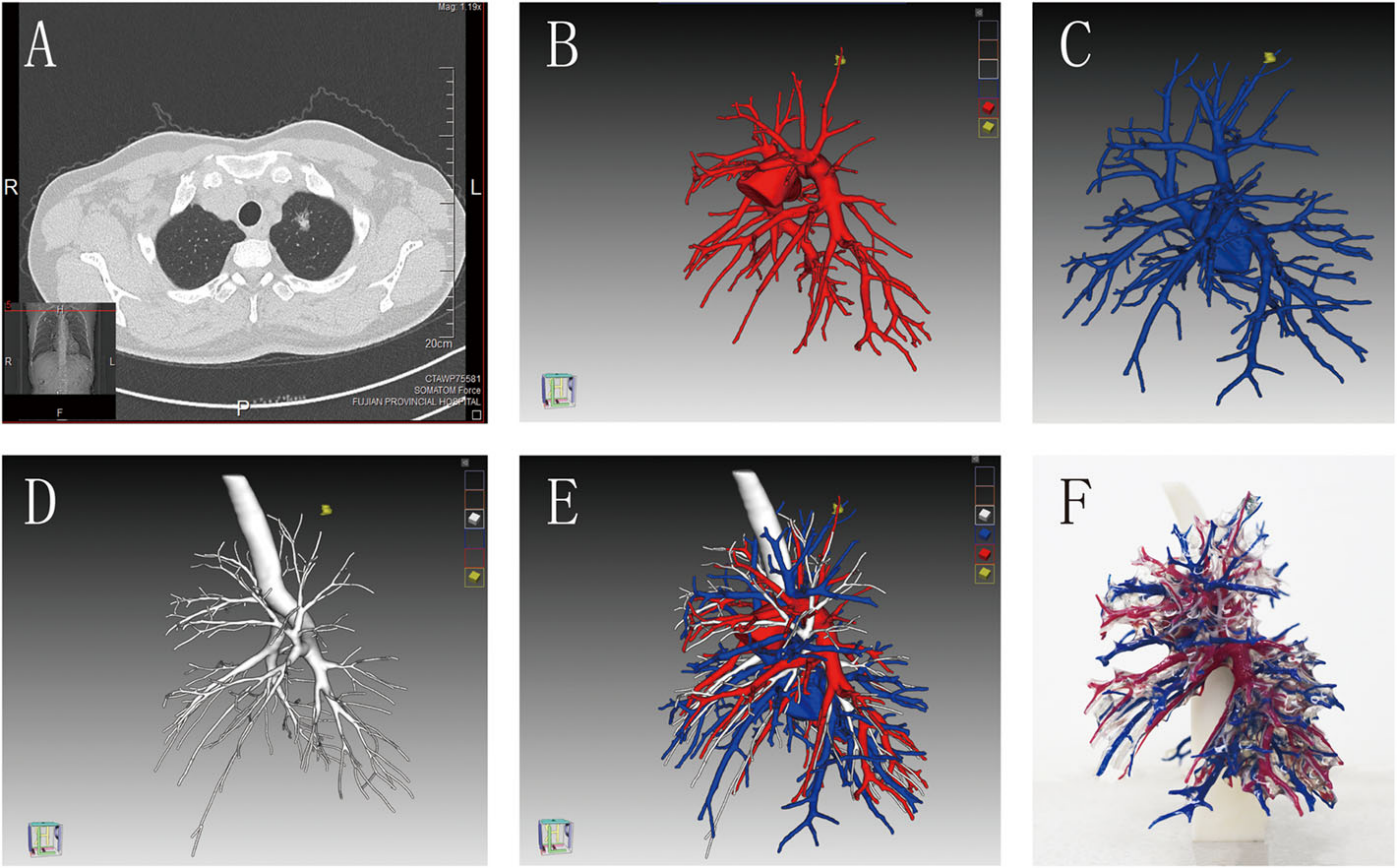
CT image, 3D reconstruction image, and 3D model of the same case. **a** The patient’s two-dimensional (2D) computed tomography (CT) image showing a part-ground glass nodule (pGGN) in the left upper lung. **b**–**e** The patient’s three-dimensional (3D) CT reconstruction image, from which the lung veins, bronchus, and arteries can be more intuitive. **f** The patient’s 3D print of the lung model not only allows the visualisation of the lung anatomy but also facilitates coordinate positioning, accurately locates the lesion, and helps in the precise wedge resection of the lesion during surgery

All patients in both groups underwent plain and enhanced chest CT scans for further diagnosis and to determine tumour location. Additionally, 3D reconstruction and 3D printing were performed for the experimental group to further locate the tumour, to determine the anatomical relationship and any anatomical variations, and to simulate the operative process. The Siemens Sensation 64-slice CT scanner with 1.2-mm pitch and 1.0-mm scanning thickness was used in this study. The contrast agent iofol, manufactured by Jiangsu Hengrui Pharmaceutical Co. Ltd. (100 mL: 74.1 g Chinese medicine quasi-word H20143027), was intravenously administered to the elbow. The arterial and venous phase images were collected 25 and 55 s after the injection of the contrast agent, respectively. The IQQA®-Chest system was used to preserve the pulmonary artery, pulmonary vein, bronchus, tumours, hilum, and swollen lymph nodes and to reconstruct these structures with a 1.50-mm thickness. The pulmonary artery was red, the pulmonary vein was blue, and the bronchus was white. The 3D model was printed with Objet1000 Plus (STRATASYS Company). The physical printing ratio was 1:1, and the printing material was photosensitive resin (Fig. 1).

### Preoperative positioning

The “coordinate positioning method” was utilised for preoperative positioning. This method is widely used for mapping and determining object positions. Generally, to determine the position of a point, the number or angle should be identified. In the plane, two axes perpendicular to each other and having a common origin to form a plane rectangular coordinate system are identified, with the horizontal and vertical axes noted as such. This method was somewhat different when determining the location of the lesion in the experimental and control groups.

**Fig. 2:**
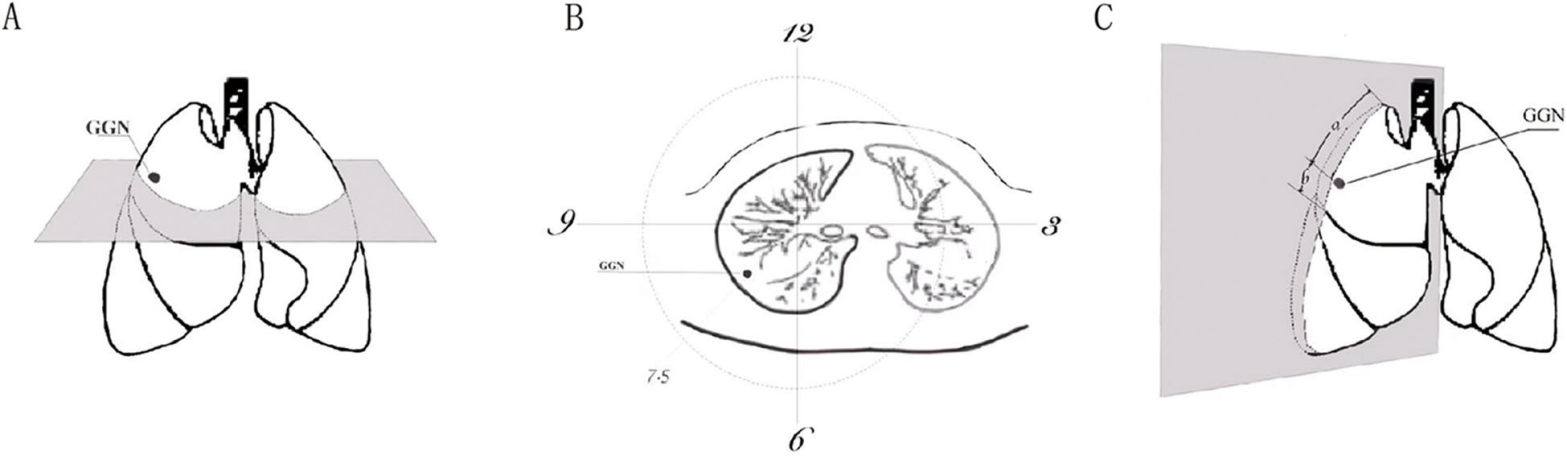
The application process of the coordinate positioning method. **a**, **b** The horizontal orientations. Using the right upper lung lesion as an example, the CT horizontal section (where the lesion is located) is selected, the right chest contour is regarded as the clock face, and the midline of the clavicle is intended to be the 12 o’clock position. The right midline is intended to be the 9 o’clock position, and the lesion in the “Clock Positioning” legend is at the 7.5 o’clock position. **c** The vertical axis orientation. Using the right upper lung lesion as an example, the axis of the 7.5 o’clock position on the 3D lung model is selected. The length of the lesion to the tip of the lung is recorded as “a”, and the length of the lesion to the interlobular fissure is recorded as “b”. The proportional position of the lesion on this axis is calculated

The horizontal axis data were derived from the measurement of the horizontal level of the lung lesion observed in the CT image. The “clock alignment method” was adopted, assuming that the horizontal plane of the right chest was a clock and the lesion was the part indicated by the hour hand. Using the right upper lung lesion as an example (Fig. 2a), the horizontal CT section (where the lesion was located) was selected, the right chest contour was regarded as the clock face, the midline of the clavicle was the 12 o’clock position, and the right midline was intended to be the 9 o’clock position (Fig. 2b). According to the “clock positioning method”, the lesion was at 7.5 points. In both groups, the horizontal axis clock positioning method can be established in the CT horizontal plane.

On the contrary, the measurement of the vertical axis adopted a “scale-localisation algorithm”. A difference was observed in the proportional positioning method between the experimental and control groups. In the experimental group, the upper end to the lower end of the lobe where the lesion was located on each longitudinal axis was considered the measurement interval. Depending on the lobes, the upper end can be the tip of the lung or the interlobular fissure, whereas the lower end can be the interlobular fissure or the base of the lung. On the 3D lung model, the distance from the lesion to the upper and lower ends was measured on the vertical axis, and the proportional position of the lesion on the vertical axis was calculated. Furthermore (with the right upper lung lesion as an example), the axis on the 7.5-h position on the 3D lung model was selected. The length of the lesion to the tip of the lung and the interlobular fissure was recorded as “*a*” and “*b*”, respectively. The proportional position of the lesion on the vertical axis was calculated using the formula: [*a*/(*a* + *b*)] (Fig. 2c).

In the experimental group A, the vertical position was located using the proportional segmentation method with the scale positioning technique, which was performed on the 3D lung model. The following steps were performed: (1) locate the lobe where the nodule was located on the 3D model, (2) measure the distance between the nodule and the upper boundary of the lobe (*a*) and the length of the upper and lower boundaries of the lobe (*a* + *b*), and (3) the ratio of the distance from the upper boundary (*a*) to the length (*a* + *b*) of the upper and lower boundaries of the lung lobe [*a*/(*a* + *b*)] was the position of the nodule in the longitudinal axis of the lobe.

In the control group B, preoperative vertical positional localisation was performed according to the sagittal and coronal planes of the CT image, using the following steps: (1) identify the lobe where the nodule was located on the CT image, (2) count the number of CT slices of the nodule from the border of the lung (*n*) and the total number of CT layers (*N*) of the upper and lower boundaries of the lobe, (3) calculate the ratio of the number of CT layers (*n*) of the nodule to the upper boundary of the lobe to the total number of CT layers (*N*) of the upper and lower boundaries of the lobe, thereby positioning the nodule longitudinally in the lobe.

In addition to the coordinate localisation method, we similarly selected anatomical landmarks, such as the apex of the lung, bottom of the lung, front edge of the lung rib, midline of the lung rib surface, posterior edge line of the lung rib surface, and the interlobular fissure as references. Maneuverer, the lung segment area, tracheal block expansion, and methods of finger touch detection, among others, were performed to accurately position the lesion.

### Surgical methods

**Fig. 3:**
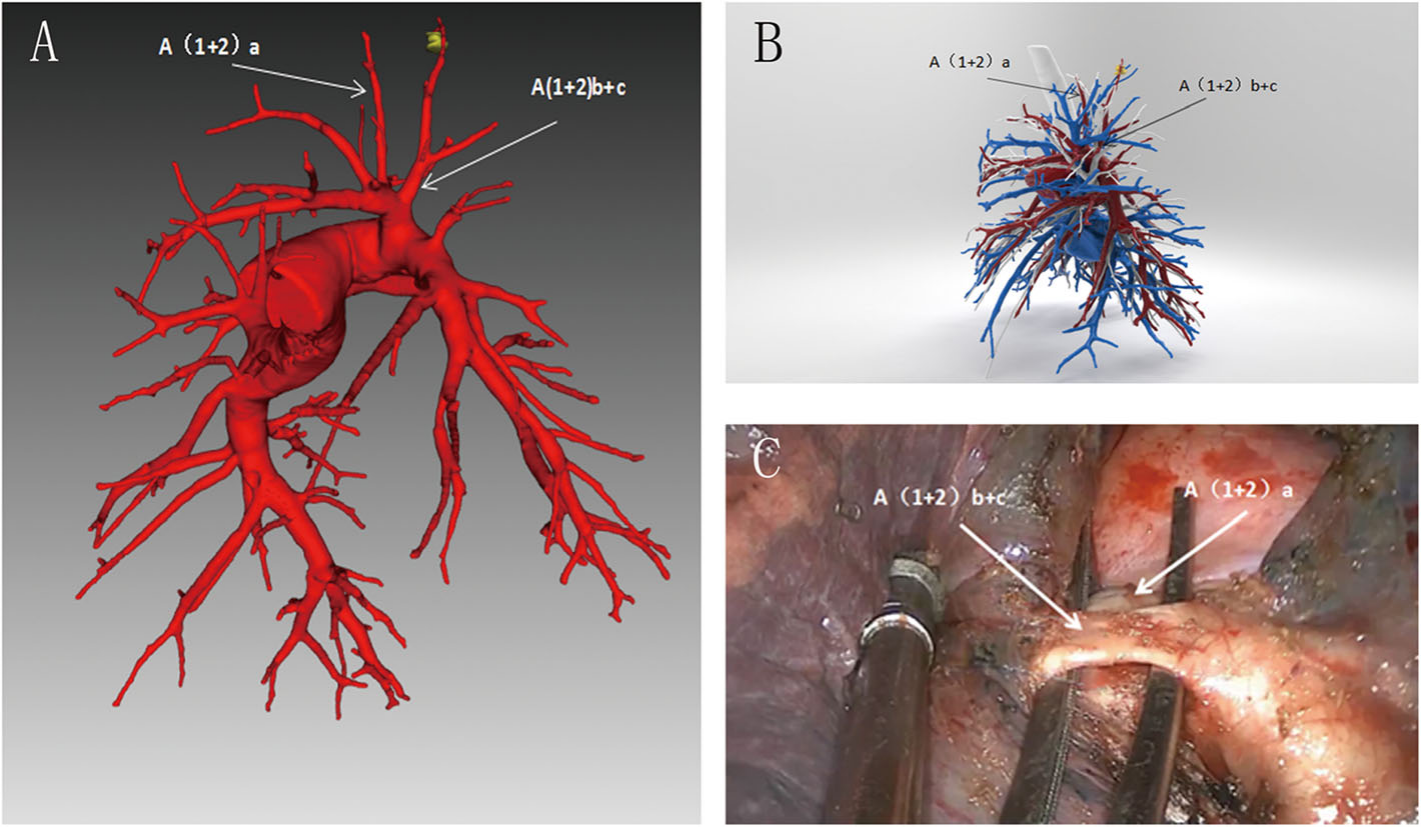
Three-dimensional reconstruction CT image of the same patient and intraoperative-related anatomy. **a** Image of pulmonary artery reconstruction, with arrows pointing to A(1 + 2)a and A(1 + 2)b + c. **b** The patient’s pulmonary vein, pulmonary artery, bronchi, and pulmonary nodules are shown in the reconstructed image. **c** The patient’s intraoperative pulmonary anatomy is consistent with preoperative 3D reconstruction and 3D printing

All patients underwent uniportal video-assisted thoracoscopic segmentectomy and systemic lymphadenectomy. The operation was performed by the treatment team led by the chief physician of the Department of Thoracic Surgery, Fujian Provincial Hospital. The assistant adopted a “same side, high position, single hand, sideway” posture mirror [6], and the operator performed the procedure using a thoracoscopic instrument. The location of the intraoperative nodules was determined using data from the preoperative positioning methods described previously. A wedge-shaped resection was initially performed and followed by an intraoperative frozen section examination. Based on the frozen section examination, the segmental vein, artery, and bronchus were separated at the anatomical level and the linear cutting suture device was broken or disconnected. To determine the inter-segment plane, the “Lung Expansion-Falling Method” [7] was utilised for the last segment of the lung fissure. The two groups were separated from the inter-segment plane of the rib surface, and a straight-section cutting stapler was used to process the inter-segment plane. During the operation, the frozen tumour and margin tissues were sent for pathological examinations and the combined lung segment or lobectomy was determined according to the distance between the tumour and the margin (2 cm). Subsequently, a conventional systemic lymph node dissection was performed. One chest tube was placed in the posterior margin of the incision, and one micro-thoracic tube was placed in the lower thoracic cavity (Fig. 3).

### Observation indicators

Both procedures were performed by the same surgeon. The operation was performed by the chief physician and assisted by two surgeons. The perioperative condition of patients was monitored by the observer. This is a single-blind study. In other words, only the patients in this study were unaware of their group allocation.

The surgical method transfer rate, operative time, intraoperative blood loss, postoperative complication rate, and segmental conversion rate of the lobectomy were measured and all these variables, except the postoperative complication rate, constituted the primary outcome measures. We also evaluated differences in the aforementioned variables between the control and experimental groups. The surgical transfer rate refers to the proportion of cases converted to open surgery due to selective reasons such as intraoperative thoracic adhesions and intraoperative bleeding. The surgical method conversion rate is the proportion of cases in which segmental resection was converted to a lobectomy due to various factors during the operation. The operative time was defined as the time from the commencement of the skin incision to the completion of suturing (*h*). Intraoperative blood loss was defined as the amount of blood absorbed by the gauze and the intraoperative suction (mL). In addition, it was determined using the weighing method to calculate the attracting liquid haemoglobin content.

### Statistical analysis

All data were analysed using SPSS21.0 statistical software. Measurement data were expressed as $\left(\overline{X}\pm s\right)$, and the two-sample *t* test was used for comparison between groups. The number of count data (*n*), and the ratio of count data (%), calculated by the *χ*^2^ test or Fisher’s exact probability method, were used in this study. *P* < 0.05 indicated a statistically significant difference.

## Results

### Surgical approach transfer rate

The surgery approach transfer rate was 0% and 10.5% for groups A and B, respectively (*p* < 0.05). Among the 51 patients in group A, none were transferred to thoracotomy. In this group, vascular variation was identified and avoided by performing the 3D CT reconstruction combined with 3D printing technology. On the contrary, among the 38 patients in the control group B, four patients underwent conversion to thoracotomy owing to the vascular variation of A1+2L, V8+9R, V4+5L, and A8+9R.

### Surgical method conversion rate

The surgical method conversion rate was 0% and 10.5% for groups A and B, respectively (*p* < 0.05). In the experimental group A, none were converted to lobectomy, and vascular variation was identified and avoided through 3D CT reconstruction combined with 3D printing technology. On the contrary, in the control group B, four patients underwent conversion to lobectomy due to the vascular variation of A4+5L, A1+2L, B6R, and lack of incision margin (< 2 cm).

### Operative time

The operative time was 2.07 ± 0.24 and 2.55 ± 0.41 h for groups A and B, respectively, showing a significant difference (p < 0.05). The difference in the operation time between the two groups was mainly due to intraoperative localisation and complications during the separation of various structures. The intraoperative localisation time was defined as the time required to determine where the lung nodules are located. Two patients in the control group underwent a wedge-shaped resection to obtain the lesion. Compared to the control group B, the duration of intraoperative positioning in the experimental group A was shortened by approximately 5–13 min; although, such a difference was not significant. The problems encountered during the separation of anatomical structures included tumour identification difficulties, bleeding, and anatomical variations.

### Intraoperative blood loss

There was a significant difference in the intraoperative blood loss volume between groups A and B (43.25 ± 13.63 mL vs. 96.68 ± 32.82 mL, *p* < 0.05). In the experimental group, vascular variation was identified and avoided through 3D CT reconstruction combined with 3D printing technology, and therefore, bleeding was reduced.

### Postoperative complications

The postoperative complication rate was 3.9% and 13.2% for groups A and B, respectively (*p* > 0.05). In group A, one patient developed cerebral infarction and another patient developed pulmonary infection. In group B, one patient experienced persistent lung leakage, two patients experienced pulmonary infections, one patient had a cerebral infarction, and one patient developed arrhythmia (Tables 2 and 3).

**Table 2:** Comparison of the observation indicators between the groups

	Surgery transfer rate (no. of cases (%))	Operative time (h, *x* ± SD)	Intraoperative blood loss (mL, *x* ± SD)	Postoperative complication rate (no. of cases (%))	Conversion rate (no. of cases (%))
Experimental Group A (*n* = 51)	0 (0)	2.07 ± 0.24	43.25 ± 13.63	2 (3.9)	0 (0)
Control group B (*n* = 38)	4 (10.5)	2.55 ± 0.41	96.68 ± 32.82	5 (13.2)	4 (10.5)
*t* value		− 6.366	− 9.447		
P value	0.030	< 0.001	< 0.001	0.132	0.030

**Table 3:** Comparison of the postoperative complications between the groups

	Persistent lung leak (no., (%))	Atelectasis (no., (%))	Pulmonary infection (no., (%))	Cerebral infarction (no., (%))	Arrhythmia (no, (%))	Total (no., (%))
Group A (*n* = 51)	0 (0)	0 (0)	1 (2.0)	1 (2.0)	0 (0)	2 (3.9)
Group B (*n* = 38)	1 (2.6)	0 (0)	2 (5.3)	1 (2.6)	1 (2.6)	5 (13.2)
*P* value	0.427	1.000	0.573	1.000	0.427	0.132

## Discussion

With the widespread use of low-dose CT scans and the increasing number of physical examinations, the number of GGNs detected early in the course of lung cancer has been increasing [8]. In recent years, with the advancement of thoracic surgical techniques, segmentectomy has replaced classic lobectomy as the treatment for small lung malignancies. Anatomical segmentectomy has gradually proven to be effective. Surgical treatment that promotes optimal lung tissue retention has an oncological effect comparable to that of lobectomy [9]. Previously, standard lobectomy was performed more often, resulting in unnecessarily extensive surgeries to remove GGNs. With the advancement of thoracic surgery in recent years, the rate of sublobar resection, especially the resection of a lung segment, has significantly increased.

This study was established based on the common problems encountered in segmental resection. The patients’ preoperative CT imaging data were used to establish the 3D imaging data of the affected lung using the 3D imaging software program, and the 3D printing technology was subsequently used to create pulmonary blood vessels. A 3D anatomical model of the lungs with important structures such as the bronchus and lesions was established. Several important indicators that are closely related to the quality of surgery, such as surgical time, intraoperative blood loss, surgical approach transfer rate, surgical method conversion rate, and postoperative complication rate, were collected to investigate the value of the 3D-printed model of the lung in segmentectomy.

The lung GGNs are usually non-solid lesions, and they are difficult to locate using the finger touch method. In clinical practice, CT-mediated injection of Meilan and the placement of coils around the lesion have been similarly used to locate small lesions that are difficult to detect. Meilan can be absorbed by the lung tissue and stays on the surface of the lungs for a short period. As such, Meilan stains often disappear during surgery, and the coils placed in the lung tissue are often difficult to handle owing to their small size and softness. Therefore, these methods are often ineffective for locating lesions.

The 3D-printed model of the lung helps the surgeon in locating the lesion during surgery. The advantages of 3D reconstruction combined with 3D printing technology compared to traditional two-dimensional (2D) and 3D positioning in CT images are obvious. With the aid of the 3D-printed model of the lung, it is easy to visually determine the area where the lesion is located; apply the coordinate positioning method; select important anatomical landmarks, such as lung boundary, lung tip, lung base, and leaf fissure, as reference points; and measure the lesion and different anatomical features from multiple angles. The corresponding distance of the marker can accurately determine the location of the lesion. In the 3D-printed model, the surgeon can intuitively calculate the distance between the nodule and the upper boundary of the lung and the length of the upper and lower boundaries of the lung, thereby reducing unnecessary errors. In 2D and 3D CT images, the distance between the nodule and the upper boundary of the lung lobe and the length of the upper and lower boundaries of the lung lobe are calculated by the number of CT layers. Given that an actual measurement cannot be performed, the error is inevitably increased during positioning. In this study, the intraoperative localisation time of the experimental group A was shorter than that of the control group B; although, such a difference was not significant (*p* > 0.05). Supposedly, this could be due to the small sample size; hence, we intend to continuously collect cases to validate this finding in subsequent studies.

The application of 3D printing technology based on 3D reconstruction can facilitate preoperative and intraoperative positioning. Depending on the attribution of blood vessels and bronchial tubes near the nodules, lung nodules can be located on the corresponding pulmonary section or even subsection. Owing to the improvement in the accuracy of lesion positioning, tissues from the lesion can be obtained with wedge excision, and the frozen section examination can be timely performed, consequently reducing the operative time. In this study, lesion removal was not successfully completed with a single attempt in two patients in the control group who first received a wedge resection; however, the lesion was obtained after an additional resection, which increased the total wedge resection time. This was not the case in the experimental group. Therefore, 3D printing technology can improve the success rate of lesion resection during intraoperative positioning.

The 3D-printed model of the lung model can help the surgeon to identify relevant anatomy and characteristic variations during the procedure. Different anatomical variations of the bronchi and blood vessels can cause serious consequences if improperly handled during thoracoscopic surgery. A clear 3D structure is difficult to achieve by exclusively using data obtained from 2D CT images, which is inconvenient for a multi-angle reference. Although 3D imaging overcomes this disadvantage, some anatomical variations are still difficult to determine. The 3D-printed model of the lung facilitates the detection of relevant anatomical variations in advance, thereby facilitating the optimising of the surgical procedure and preventing accidental injury to arteries, veins, and bronchi. Among 51 patients in the experimental group A, none were transferred to thoracotomy. In this group, vascular variation was identified and avoided by performing the 3D CT reconstruction combined with 3D printing technology. On the contrary, among 38 patients in the control group B, four patients underwent conversion to thoracotomy owing to the vascular variation of A1+2L, V8+9R, V4+5L, and A8+9R. Thus, 3D CT reconstruction combined with 3D printing technology reduces the surgical method conversion rate. As such, the anatomical variation can be mastered before surgery and measures can be taken not only to prevent excessive bleeding during surgery but also to avoid remediation of anatomical structure damages. Therefore, a comprehensive understanding of the patient’s detailed anatomy before surgery is crucial in preventing surgical complications.

In this study, no statistically significant difference in the postoperative complication rate was observed between the two groups, which was comparable to the results obtained by Cheng et al. [10]. However, such an observation can also be attributable to the sample size used in this study. Thus, we intend to continuously increase the sample size and in the future, we aim to obtain more comprehensive data. In theory, the incidence of postoperative complications in the experimental group A should have been lower than that of the control group B. Along with reduced surgical bleeding, surgical time, surgical injury, and surgical conversion rate, as well as anaesthesia and mechanical ventilation time during surgery, should have been shorter in the experimental group A than in the control group B. Additionally, a reduced time would similarly be observed for lung collapse, and all of these factors would have reduced the incidence of lung tissue injuries and postoperative complications.

Nevertheless, this study has a few limitations. The current sample size is limited by research funding, which led to the insufficient sample size and the latent deviations of our results. If possible, we intend to further expand the sample size in subsequent experiments to meet the sample size requirements. Another limitation is the single-blind design of the trial in this study. As a result, a subjective bias is probable, which may lead to treatment imbalance between the experimental and control groups.

## Conclusions

The application of 3D chest CT reconstruction combined with 3D printing technology in segmental resection can reduce the surgical conversion rate, operative time, intraoperative blood loss, and segmental lobectomy conversion rate. This technique will facilitate the improvement of surgical accuracy and safety, and thus, it is worth applying in clinical settings.

## Supplementary information

**Additional file 1:.** CONSORT Flow Diagram

## Data Availability

The datasets used and/or analysed during the current study are available from the corresponding author on reasonable request.

## Abbreviations

CT: Computed tomography
GGNs: Ground glass nodules
3D: Three-dimensional
NCCN: National Comprehensive Cancer Network
2D: Two-dimensional
