# Supplementary material for: Three-dimensional printing technology for localised thoracoscopic segmental resection for lung cancer: a quasi-randomised clinical trial
Source: World J Surg Oncol. 2020 Aug 24;18:223. doi: 10.1186/s12957-020-01998-2 (PMC7446226; doi:10.1186/s12957-020-01998-2)
Supplement: Supplementary file 1 — Additional file 1:. CONSORT Flow Diagram [file 12957_2020_1998_MOESM1_ESM.doc]

**CONSORT Flow Diagram**

**Allocation**

**Analysis**

**Follow-Up**

**Enrollment**

From March 2016 to September 2018, planned to operated segmentectomy in Fujian Provincial Hospital. Meet the Inclusion criteria.(n=101)

Excluded

refused to involve (n=4)

poor physical condition (n=2)

cancelled surgery unexpected (n=1)

Analysed (n= 51)
♦ Excluded from analysis (n=0)

Lost to follow-up (give reasons) (n= 0)

Discontinued intervention (Rapid freezing pathology suggest benign lesion and underwent wedge resection only) (n=3)

Allocated to intervention (n=54)

♦ Received allocated intervention (n=54)

♦ Did not receive allocated intervention (n=0)

Lost to follow-up (give reasons) (n= 0)

Discontinued intervention (Rapid freezing pathology suggest benign lesion and underwent wedge resection only) (n=2)

Allocated to intervention (n= 40)

♦ Received allocated intervention (n=40)

♦ Did not receive allocated intervention (n=0)

Analysed (n=38)
♦ Excluded from analysis (n=0)

Randomized (n= 96)
